# Development of a novel alpha7-nicotinic acetylcholine receptor-selective cell-penetrating peptide for intracellular cargo transport

**DOI:** 10.1080/10717544.2025.2587378

**Published:** 2025-11-30

**Authors:** Lahra Weber, Brittany C. V. O’Brien, Maegan M. Weltzin

**Affiliations:** a Department of Chemistry and Biochemistry, University of Alaska Fairbanks, Fairbanks, AK, United States

**Keywords:** Cell penetrating peptide, drug delivery, alpha 7, nicotinic acetylcholine receptor, target selectivity, intracellular cargo delivery, rabies virus glycoprotein

## Abstract

Cell membranes present barriers to the intracellular delivery of therapeutic agents. This impediment is frequently exacerbated by the hydrophobic characteristics of many such molecules, ultimately reducing the efficiency of their cellular uptake and therapeutic effectiveness. Therapeutics are being created that exploit natural bypass mechanisms by forming complexes with cell-penetrating peptides (CPPs) derived from viruses. However, current CPPs lack the ability to selectively target precise cellular macromolecules. As a result, they are distributed broadly and cause off-target side effects. Neurotropic CPPs derived from the rabies virus glycoprotein (RVG) can access the brain by binding to plasma membrane targets, including, but not exclusively, nicotinic acetylcholine receptors (nAChRs). To overcome this barrier of minimal target selectivity, we designed several chimeric peptides composed of regions from the RVG and *α*-bungarotoxin, an α7 subtype-selective protein. Using human nAChRs expressed in *Xenopus laevis* oocytes, we screened the selectivity of our peptides using two-electrode voltage clamp electrophysiology. We identified a peptide with improved α7 nAChR subtype selectivity and apparent potency compared to the control RVG peptide. Using mammalian Neuro-2a cells, we demonstrated that our peptide depends on α7 nAChR plasma membrane expression to internalize and carry small-molecule payloads into neuronal-like cells without significant cytotoxic effects. Our novel α7 nAChR subtype-selective CPP may be useful in research applications requiring cargo delivery. Translationally, our α7 nAChR-selective CPP holds potential to be a dual drug delivery system to transport cargo into the brain for the treatment of neurological diseases.

## Introduction

Biological cell membranes are natural barriers for living cells and are the main hurdles in the efficient delivery of bioactive and therapeutic agents, often due to the hydrophobic nature of many bioactive molecules (Nam et al. 2023). To increase the delivery of therapeutics, cell-penetrating peptides (CPPs) can be conjugated to drugs to increase cytosol access. The use of CPPs, which are often nontoxic, can enhance the passage of payloads like nanoparticles, silencing RNA (siRNA), DNA, and other compounds. However, CPPs generally interact broadly with cellular macromolecules and lipid bilayers, resulting in uncontrolled cargo delivery, undesired side effects, and ultimately failing to alleviate the disease burden. One approach to avoid this pitfall is the engineering of target-specific CPPs. In this approach, improving cargo delivery can be achieved by incorporating a CPP that selectively recognizes unique cell surface macromolecules that are expressed in specific tissues or microenvironmental cues found predominantly during disease states. The selective honing drastically reduces off-target drug accumulation, minimizes adverse effects, and increases the therapeutic window.

One specific approach for the development of CPPs is the use of viral glycoprotein components, as these interact with host targets expressed on cell membranes to facilitate virion endocytosis (Jiao et al. 2009; Schnell et al. 2010; Rizzuti et al. 2015; Okafor 2025). To exploit the neurotropic ability of the rabies virus, small CPPs have been created from its glycoprotein, and have been used to deliver functional cargo into the murine brain within 24 hr (Jia 2021; Gong et al. 2012; Kumar 2007; Huey et al. 2017; Fu et al. 2018; Yang 2024). While this development is an important stepping stone for drug delivery into the brain, CPPs derived from the rabies virus glycoprotein so far lack target specificity (O’Brien 2024). The rabies virus glycoprotein is known to interact with host proteins, including the neural cell adhesion molecule (Kadmon et al. 1990; Thoulouze et al. 1998), p75 neurotrophin receptor (Tuffereau et al. 1998; Gluska 2014), metabotropic glutamate receptor subtype 2 (Wang 2018), integrin β1 (Shuai 2020), and nicotinic acetylcholine receptors (nAChRs) (O’Brien 2024; Lian et al. 2022; Hueffer 2017). We have recently demonstrated that a rabies virus glycoprotein-derived peptide (RVG) similar in sequence to that of other RVG CPPs (Jia 2021; Kumar 2007) inhibits the function of the α7 nAChR via a competitive mechanism, in addition to heteromeric subtypes, albeit less efficaciously (O’Brien 2024). These data demonstrated that RVG CPPs have functional actions independent of the attached cargo and, therefore, need to be considered for therapeutic applications.

nAChRs are pentameric ligand-gated ion channels that are initially activated in response to the neurotransmitter acetylcholine (ACh) to mediate cholinergic tone within the brain. Through combinations of the neuronal α2-7 and β2-4 subunits, diverse populations of homomeric and heteromeric subtypes are expressed in the CNS (Millar and Gotti 2009; Shan 2024), with the α4β2 and α7 subtypes having the greatest abundance in the mammalian brain (Colombo et al. 2013; Millar and Harkness 2008; Morioka 2014). While the α7 nAChR is found predominantly on neurons, it has also been identified in astrocytes and microglia (Liu 2012; Morioka 2015) and in the mitochondria of several cell types (Nakamura et al. 2025; Gergalova 2012). In the CNS, α7 nAChRs are enriched in the hippocampus and prefrontal cortex, areas of the brain responsible for learning, memory, and attention (Levin 2012; Leiser et al. 2009). Dysregulation or dysfunction of this subtype is associated with neurological diseases, including Alzheimer's disease, Parkinson's disease, schizophrenia, nicotine addiction, and major depressive disorder, making this subtype a therapeutic target (ElNebrisi et al. 2025; Schaaf 2014; Gillentine 2017; Brunzell and McIntosh 2012; Mineur et al. 2018; Magnussen 2025; Koukouli 2025). High affinity and nAChR subtype selectivity are obtainable with naturally occurring peptides found in the venom of animals (Nirthanan 2020; Nys 2022; Bekbossynova et al. 2021; Barber et al. 2013; Gulsevin 2021). *α*-bungarotoxin (*α*-btx) is a venom component from the elapid Taiwanese banded krait snake (*Bungarus multicinctus)*. This ‘three-finger protein’ has three loops, with loop 2 (lp2) interacting predominantly with the α7 nAChR orthosteric binding pocket. Devising chimeric peptides that utilize the existing high-affinity *α*-btx and RVG CPP may be a new avenue for increasing target selectivity.

We sought to harness the endogenous selectivity of the snake toxin *α*-btx with components of the rabies virus glycoprotein that have been used previously as a CPP, especially amino acid residues 174–203 (RVG), to generate an α7 nAChR-selective CPP (Weber et al. 2025). To accomplish this, we developed and screened a series of chimeric peptides on 10 nAChR subtypes and isoforms to evaluate selectivity using two-electrode voltage clamp (TEVC) electrophysiology. The cytotoxicity profile, cargo delivery, target selectivity, and CPP capabilities were assessed using the mammalian N2a cell line, which has a neuronal phenotype. Our results demonstrate that we have engineered a novel α7 nAChR selective CPP, a first of its kind, that has concentration-dependent antagonist activities, is not cytotoxic, and can transport cargo into neuron-like cells. Clinically, our target-selective CPP may be advantageous, as it could be used as a dual-acting drug delivery system to transport therapeutic cargo into α7 nAChR-expressing cells.

## Materials and methods

### Reagents

Acetylcholine chloride (ACh, cat. # A6625), atropine sulfate (cat. # A0257), bovine serum albumin (BSA, cat. # A7030), dimethylsulfoxide (DMSO, cat. # D8418), mecamylamine hydrochloride (Meca, cat. # M9020), and methyllycaconitine citrate salt (MLA, cat. # M168) were purchased from Sigma Aldrich (St. Louis, MO). All other buffer reagents were purchased from Sigma Aldrich unless otherwise specified. Chimeric peptides were designed by M. M. Weltzin (see Table 1 for sequences) and purchased from Elim BioPharmaceuticals (Hayward, CA) with HPLC purity >90%. The lyophilized peptides were stored in the dark at −20°C until use. Peptides were first dissolved in DMSO before dilution with the appropriate media or buffer. Drug solutions were made fresh daily and diluted as needed.

**Table 1.** Sequence alignment of the RVG peptide and *α*-btx lp2 peptide. Bolded residues indicate sequence homology. Each peptide region (R) was tested and used to create the chimeric peptides.

Virus/Toxin	Region 1	Region 2	Region 3
RVG	YT-IW	MPENPRLGTS	CDIFTNSRGKRASKG203
*α*-btx	YRKMW	–	CDAFCSSRGKVVELG43
RRA	YT-IW	MPENPRLGTS	CDAFCSSRGKVVELG29
ARA	YRKMW	MPENPRLGTS	CDAFCSSRGKVVELG30
ARR	YRKMW	MPENPRLGTS	CDIFTNSRGKRASKG30

Eagle's Minimum Essential Medium (EMEM, cat. # 30−2003), fetal bovine serum (FBS, cat. # 1500−500), Penicillin-Streptomycin (cat. # K952), and pH 7.4 phosphate-buffered saline (PBS, cat. # 114−056−101) were purchased from VWR International, LLC (Radnor, PA). Both Gibco Opti-Minimal Essential Medium (Opti-MEM, cat. # 31985070) and Trypsin-EDTA (0.25%, cat. # 25200056) were purchased from Gibco ThermoFisher Scientific (Waltham, MA). Lipofectamine 2000 Transfection Reagent (cat. # 11668019) and alamarBlue Cell Viability Reagent (cat. # DAL1025) were purchased from Invitrogen (ThermoFisher Scientific).

### DNA constructs and cRNA synthesis

As described in our previous works (O’Brien 2024; O’Brien et al. 2023), individual human nAChR subunits (α3 (NM_000743.5), α4 (NM_000744.5), α5 (NM_000745.3), β2 (NM_000748.2), β4 (NM_000750.5), and α7 (NM_000746.3) were generous gifts from Drs. Ronald J. Lukas and Paul Whiteaker (Barrow Neurological Institute, Phoenix, AZ). Also received was an α6/α3 chimeric subunit,which is comprised of the α6 extracellular domain and the α3 transmembrane and intracellular domain, and the linked subunit concatenated receptor β3α6β2α4β2. The α6/α3 chimeric subunit has increased expression compared to naturally occurring α6 subunits,while maintaining α6-like pharmacology (Kuryatov et al. 2000). The β3α6β2α4β2 receptor was concatenated using 6–12 alanine, glycine, and serine repeats to link the subunits together and therefore enforcing the subunit positioning (Kuryatov and Lindstrom 2011). Each nAChR subunit cDNA was received in the mammalian expression vector pCI (Promega, Madison, WI), except for the α7 nAChR, which was in pSHE (a modified pGEMHE vector), and the concatenated receptor in pSGEM. The α7(345–348A) nAChR construct has residues 345–348 mutated to alanine, which abolishes interactions with Gαq and Gβγ without eliminating receptor expression or *α*-btx binding (King et al. 2015). α7(345–348A) DNA was synthesized from Twist Bioscience (San Francisco, CA) and inserted into the pSHE vector using XbaI (cat. # R0145) and EcoRV (cat. # R3195) restriction enzymes (New England Biolabs [NEB], Ipswich, MA). cDNA amplification was accomplished via transformation with NEB® 5-*α* competent *E. coli* cells (cat. # C2987H, NEB) and extracted using Qiagen QIAprep Spin Miniprep kits (cat. # 21706, Valencia, CA). The extracted DNA was verified via restriction enzyme digestion. The constructs in the pCI vector were verified with the enzymes XbaI and NotI (cat. # R3189), while constructs in pSHE/pSGEM were confirmed using XbaI and EcoRV. The digested DNA samples were visualized on a 1% agarose gel stained with ethidium bromide.

Before cRNA synthesis, the cDNAs were linearized using restriction enzymes (SwaI (cat. # R0604) for pCI and NheI (cat. # R3131) for pSHE/pSGEM constructs). The linearized DNA was then treated with proteinase K (cat. # P8107, NEB) and purified using the Qiagen PCR clean-up kit (cat. # 28106). The T7 mMESSAGE mMACHINE™ High Yield Capped RNA Transcription Kit (cat. # AM1344, Ambion, Austin, TX) was used to transcribe cRNA. Dual methods were employed to verify cRNA purity, including quantification by the Thermo Scientific NanoDrop 2000 spectrophotometer and electrophoresis gel imaging. cRNAs for each subunit or concatenated construct were sub-aliquoted and stored at −80°C.

The α7 pcDNA 3.1(+) and NACHO plasmid DNAs were prepared and verified as previously described in O’Brien et al. (2023). The chaperone protein NACHO DNA plasmid in pREP9 was generously gifted by Dr. R. Loring (Northeastern University, Boston, MA). To facilitate robust expression of α7 nAChRs in the mammalian N2a cell line, the α7 subunit DNA was removed from the pSHE vector and subcloned into pcDNA 3.1 (+). The final DNA construct was verified using XbaI, NotI, and PvuI (cat. # R3150) restriction enzymes (NEB). To achieve transfection-grade, endotoxin-free constructs suitable for cell culture experiments, α7 nAChR and NACHO plasmid DNAs were extracted from 10-beta competent *E. coli* cells (High Efficiency, cat. # C3019H, NEB) were grown in Circle Grow broth media (cat. # 1130001112, MP Biomedicals, Santa Ana, CA) supplemented with 0.1 mg/mL ampicillin using the EndoFree Plasmid Maxi Kit (cat. # 12362, Qiagen). The cDNA concentration and purity (260/280 ratio) were measured using the NanoDrop 2000 spectrophotometer. cDNAs were stored at −20°C until use.

### Oocyte preparation and cRNA injection

Expression of nAChRs was accomplished via cRNA injection into stage IV and V *Xenopus laevis* oocytes (Ecocyte Bioscience, Austin, TX). All efforts were made to minimize animal suffering and reduce the number of animals used. Homomeric α7 and concatenated β3α6β2α4β2 nAChRs were expressed by microinjection of a single cRNA transcript (40 ng/oocyte). All other heteromeric nAChR subtypes were expressed via injection of mixed-subunit cRNAs (in ng) (α4β2α5- 2.5 α4: 2.5 β2: 25 α5; α6/α3β2β3- 12 α6/α3: 12 β2: 6 β3; (α4β2)_2_α4- 12.5 α4: 0.125 β2; (α3β2)_2_α3 and (α3β4)_2_α3- 30 α: 1 β; (α3β2)_2_β2 and (α3β4)_2_β4- 1 α: 30 *β*. Each oocyte received a total of 81 nL of cRNA via impalement using a pulled glass micropipette (Drummond Scientific Company, Broomall, PA) with an outer diameter of ~40 µm. Oocytes were incubated in buffer (82.5 mM NaCl, 2.5 mM KCl, 1 mM MgCl_2_, 1 mM CaCl_2_, 1 mM Na_2_HPO_4_, 5 mM HEPES, 600 μM theophylline, 2.5 mM sodium pyruvate, 50 μg/mL penicillin, streptomycin, neomycin, or gentamycin sulfate, pH 7.5, using NaOH) at 13°C for 72–120 hour prior to recording, with daily buffer changes.

### Electrophysiology

nAChR function was evaluated using two-electrode voltage clamp (TEVC) electrophysiology. At no less than 72 hour post-cRNA injection, *Xenopus laevis* oocytes were voltage clamped at −70 mV using an Axoclamp 900 A amplifier with pClamp 10.6 software (Molecular Devices, LLC, Sunnyvale, CA) for data acquisition and analysis. DC offset was accomplished using a 40 Hz high-pass filter and a 10 kHz low-pass Bessel filter. Recording electrodes were pulled from thin-wall capillary glass (World Precision Instruments, Sarasota, FL) and filled with 3 M KCl. The electrode resistance ranged from 0.5–10 MΩ. Oocytes with leak currents below −100 nA were discarded.

Using a 16-channel, gravity-fed, perfusion system with automated valve control (AutoMate Scientific, Inc., Berkeley, CA), drug solutions were applied to voltage-clamped oocytes. Drug solutions were made in an Oocyte Ringer 2 (OR_2_) recording buffer (92.5 mM NaCl, 2.5 mM KCl, 1 mM MgCl_2_•6H_2_O, 1 mM CaCl_2_•2H_2_O, 5 mM HEPES, pH adjusted to 7.5 using NaOH) containing 0.1% BSA and 1.5 µM atropine sulfate. BSA was used to prevent peptides from sticking to plastics in the experimental apparatus, and atropine sulfate was used to block responses from potential endogenously expressed muscarinic receptors (Weltzin et al. 2019; Weltzin et al. 2016; Whiteaker 2007; Luo 2014). Owing to the reduced OR_2_ solubility of some peptides, all the peptides were initially solubilized in 100% DMSO for 5 min and then diluted with OR_2_ to the desired concentration, resulting in a final DMSO concentration of 2% in all the peptide solutions. For each nAChR subtype used in this study, 2% DMSO did not affect peak responses evoked by the ACh EC_90_ (data not shown). Solutions are made fresh daily.

It is well appreciated that varying cRNA subunit injection ratios can result in the expression of a mixture of nAChR isoforms. Based on the data from our previous work (O’Brien 2024; O’Brien et al. 2023), where each subtype/isoform is expressed separately, the effective concentration 90 (EC_90_) was calculated (α4β2α5 12 µM; (α4β2)_2_β2 18 µM; concatenated β3α6β2α4β2 26 µM; α6/α3β2β3 40 µM; (α3β2)_2_β2 79 µM; (α3β4)_2_β4 220 µM; (α3β4)_2_α3 410 µM; (α4β2)_2_α4 1.16 mM; α7 1.30 mM; and (α3β2)_2_α3 3.05 mM).

The potential subtype selectivity of each tested peptide was determined using an nAChR subtype screening TEVC protocol. Table 1 displays the peptide sequence information. For each tested peptide, EC_90_ ACh was applied for 1 s to individual oocytes expressing the intended nAChR. Following an 84 s OR_2_ wash, 100 µM peptide was preapplied for 30 s, followed by a 1 s ACh EC_90_ application. The peptide-altered response was normalized to the initial ACh EC_90_ response without peptide preapplication to determine the percent inhibition caused by the peptide. Due to a priming effect of α7 nAChRs, two ACh EC_90_ hits were performed prior to inhibition with the peptides, and peptide-inhibited responses were normalized to the second ACh EC_90_ response.

For peptides that showed high α7 nAChR subtype selectivity, the apparent potency was determined. Using α7 nAChR-expressing oocytes, ACh EC_90_ was applied twice, with 225 s OR_2_ washing in between each application, as this subtype requires pre-exposure to ACh before the full response is evoked. Next, increasing concentrations of each peptide (0.01–300  µM) were preapplied for 30 s, followed by 1 s of 1300 µM (EC_90_) ACh, followed by 225 s of OR_2_ recording buffer between each drug application. The responses were normalized to the second ACh EC_90_, peptide naïve evoked response, and the resulting data were fit using an unconstrained monophasic sigmoidal inhibition equation ([Inhibitor] vs. response – variable slope (four parameters) with GraphPad Prism v.10 (Boston, MA) software.

To characterize the specific class of positive allosteric modulation that ARA mediates, comparative experiments using the known nAChR positive allosteric modulator (PAM) PNU−120596 were performed. PNU−120596 has been proven to act as a type II PAM on α7 nAChRs by recovering desensitized receptors. To confirm this, experiments were performed where α7 ACh EC_90_ (1300 µM) was coapplied with PNU−120596 following either 30 s of PNU−120596 only application or 30 s of ACh EC_90_ application to reach steady-state receptor desensitization. The same protocol was followed, in which 3 µM PNU was replaced with 0.3 µM ARA to determine whether ARA exhibited the same type of PAM activity as PNU−120596.

We observed that higher concentrations of ARA (≥10 µM) produced an outward current when applied to α7 nAChR-expressing oocytes (but not when uninjected oocytes were used). The presence of this outward current precluded us from analyzing the response data using the net-charge response as used by others (Papke and Porter Papke 2002; Horenstein 2016). Experiments were performed with the known nAChR antagonist MLA in an effort to block these currents. An MLA concentration of 10 nM was determined to be required to overcome ACh stimulation. Experiments were then performed where either 10 nM MLA or 100 µM ARA was preapplied for 10 s, followed by 10 s of coapplication. These experiments were also performed with PNU−120596 (3 µM) or Meca (10 µM) in place of MLA. We then assessed whether the outward current was due to activation of a signaling cascade that involved the α7 nAChR metabotropic activities (King et al. 2015; Sinclair and Kabbani 2023; Boesgaard 2020). Initially, we attempted to express α7(345–348A) nAChRs, a mutant that does not interact with Gαq and Gβγ (King et al. 2015), but the receptors did not express or were not functional in oocytes. Finally, we attempted to rectify the outward current by utilizing the compound YM−254890, which is known to inhibit the G-protein activity of the α7 nAChR (Boesgaard 2020). As YM−254890 needs time to penetrate cells, we added 2–20  µM to the oocyte buffer solution for 1–24 hour before applying 100 µM ARA for 15 seconds.

To characterize the ARA peptide's mechanism of antagonism, we generated ARA and ACh co-application concentration-response profiles. This was accomplished by coapplying increasing concentrations of ACh (0.010 µM–10 mM) with a single peptide concentration (0 µM, 10 µM or 100 µM) for 1 s with an 84 s wash of OR_2_ recording buffer between each drug application. Following the coapplication responses, two applications of I_max_ ACh (10 mM) without peptide were performed. The first and second ACh-only responses were comparable in amplitude, indicating that the washout time was sufficient to remove any residual ARA peptide. The coapplication data were normalized to the second ACh I_max_ response. The resulting data were fit using a monophasic sigmoidal equation ([Agonist] vs. response – Variable slope (four parameters) using GraphPad Prism v. 10 software.

TEVC experiments were conducted on at least two batches of synthesized cRNAs and at least three oocyte isolations from individual frogs. The number of experimental replicates from individuals is indicated by *N*, followed by the number of individual oocytes (*n*).

### Expression of α7 nAChRs in cultured N2a cells

Mouse neuroblastoma Neuro-2a (N2a) cells were purchased from the American Tissue Culture Collection (cat. # CCL−131, ATCC, Manassas, VA). N2a cells were maintained in EMEM supplemented with 10% FBS and 1X penicillin-streptomycin at 37°C and 5% CO_2_ in a humidified cell culture incubator. The cells were subcultured onto either poly-D-lysine-coated glass coverslips (cat. # P35GC−0−10-C, MatTek, MA) or 48-well plates (cat. # 10062−898, VWR, Radnor, PA) and transiently transfected when the dishes were approximately 70% confluent with the cells. Lipofectamine 2000 transfection reagent was used for transient transfection according to the manufacturer's protocol. The mammalian α7 nAChR-encoding plasmid DNA and the NACHO chaperone plasmid DNA were combined at a 4:1 ratio in Opti-MEM for α7 nAChR-positive groups. We previously used and proven this technique to transfect N2a cells with α7 nAChRs by (O’Brien (2024). As N2a cells endogenously express low levels of α7 nAChR, we knocked down α7 nAChR expression by transfecting 100 pmol of α7 siRNA (Thermo Fisher, 4390816) with Lipofectamine 2000 (as described above) and used these cells as our α7 nAChR knockdown control.

### N2a corrected total cell fluorescence analysis

To determine corrected total cell fluorescence (CTCF) with ImageJ (National Institutes of Health, Bethesda, MD), we calculated the mean integrated density of fluorescence in each group, using non-deconvoluted static images. To reduce bias, the observer was blinded to the fluorescence channels and free-hand traced cells that appeared healthy in size and shape in the phase channel before fluorescence was measured. The area, mean, and integrated density were measured in the fluorescence channel. Three distinct circular ROIs surrounding each selected cell were drawn to measure background fluorescence. Using these values, CTCF was calculated according to the following formula: CTCF = integrated density — (area of cell × mean background fluorescence) (Fitzpatrick n.d.). This process was repeated for four cell passages and transfections (*N*) with 30–50 cells (*n*) for each N.

### Cell viability assay

To determine if the novel peptides were cytotoxic, we assayed cell viability using the alamarBlue cell viability reagent, as we have previously performed (O’Brien et al. 2023). Briefly, N2a cells were seeded on 48-well plates, and α7 nAChR-positive groups were transfected as described in the *Expression of α7 nAChRs in cultured N2a cells* section. Twenty-four hours after transfection, the cells were exposed to different concentrations of RVG, *α*-btx lp2, or ARA (0.03–100 µM) and incubated for an additional 24 hours. 15% DMSO was added to control wells in lieu of peptide to serve as a positive control for cell death. The media was changed after 24 hour, and alamarBlue Cell Viability reagent was added to all the wells at 1/10^th^ of the volume, according to the manufacturer's instructions. After 3 hour, a Tecan Spark Multimode Microplate Reader (Tecan U.S., Inc., Morrisville, NC) was used to measure changes in fluorescence (excitation: 555 nm, emission: 600 nm, bandwidth: 20 nm). Fluorescence was measured by taking 8 × 8 measurements per well while keeping a 1500 μm border to the well walls to reduce the possible detection of fluorescence transmitted from neighboring wells or interference from well walls. For the cell viability experiments, large *N* denotes the number of 48-well plates, whereas small *n* designates the number of wells.

### Fluorescent confocal microscopy

To verify the positive transfection of α7 nAChR DNA, 80 nM Alexa Fluor 647-conjugated *α*-btx (*α*-btx-AF647, cat. # B35450, ThermoFisher) was added to EMEM growth medium with FBS and antibiotics 24 hour post-transfection and incubated for an additional 24 hour before being imaged. To determine if the RVG or ARA peptides targeted α7 nAChRs expressed in the plasma membrane of N2a cells, 30 µM FITC-tagged RVG or FITC-tagged ARA was added to the growth medium 24 hour post-α7 nAChR DNA transfection and incubated for an additional 24 hour. Peptide concentrations used were based on their functional potency as presented in *The α-bungarotoxin/RVG chimeric peptide, ARA, is highly selective for the α7 nAChR subtype,* results section. Cells were then rinsed three times with PBS before being imaged on the Olympus (Center Valley, PA) Fluoview FV10i Laser Scanning Confocal Microscope (AF647: 653 nm excitation, 668 nm emission wavelengths; FITC: 495 nm excitation, 519 nm emission wavelengths). Captured images were used for visualization and fluorescence CTCF quantification as described in the *N2a-corrected total cell fluorescence analysis* section.

### Confocal image processing

After capture, images used for visualization were only deconvoluted using ImageJ and the DeconvolutionLab 2 and PSF Generator plug-ins (Biomedical Imaging Group, École polytechnique fédérale de Lausanne). Point spread functions (PSFs) were calculated using the PSF generator and microscope settings as described in the above sections. Using the calculated PSFs and the DeconvolutionLab2 plug-in, images were deconvoluted using the Richardson‒Lucy algorithm with 10 iterations (Laasmaa et al. 2011).

To determine whether *α*-btx-AF647 or the ARA peptide is internalized into cells, we generated three-dimensional representations of cells labeled with either construct. Single or small clusters of visually healthy, labeled cells were chosen for 3D visualization and imaged at the above-mentioned microscope settings with a 1 µm slice size. The image stacks of each channel were first separated from one another, then deconvoluted separately and developed into 3D projections using the 3D project function with interpolation. The phase and fluorescence channels were then combined into one image to create a multichannel 3D model. Internalization of RVG-FITC has been previously published by O’Brien (2024).

### Data analysis

All quantified data were analyzed using GraphPad Prism v.10. Data sets comparing two groups were analyzed using Welch's *t* test. For data sets with three or more groups, one- or two-way ANOVA was performed based on the number of independent variables. The data are displayed as the means ± S.D. Throughout all the statistical analyzes, the significance levels are denoted as follows: ^ns^*P* > 0.05, *P < 0.05, **P < 0.01, ***P < 0.001, and ****P < 0.0001.

## Results

### The *α*-bungarotoxin/RVG chimeric peptide, ARA, is highly selective for the α7 nAChR subtype

**Figure 1.**
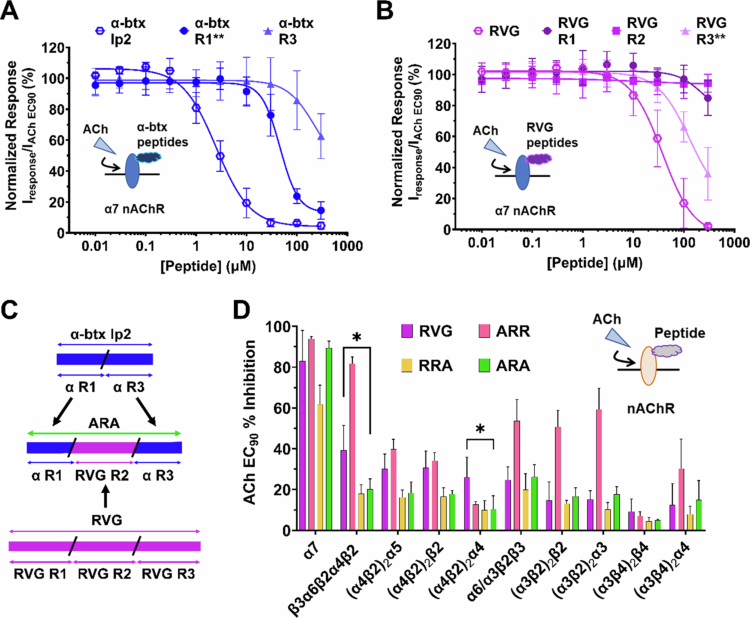
Chimeric peptides generated from regions of RVG and *α*-btx lp2 have differing selectivity profiles. TEVC analysis of the concentration-dependent effects of the test peptides on nAChR-expressing oocytes. (A) *α*-btx lp2 is highly potent (IC_50_ = 2.5 µM (CI 2.0, 3.1 µM) on the α7 nAChR (*N* = 3–4, *n* = 5–7) while the fragments are not (**P = 0.0013, Welch's t test). (B) RVG components R1, R2, and R3 are not very potent for the α7 subtype (*N* = 3–4, *n* = 5–8) compared to the full RVG peptide (**P = 0.0071, Welch's t test). (C) Schematic illustration of chimeric peptide design and nomenclature. (D) In comparison to the RVG, ARA increases the inhibition of α7 nAChR (83% ± 15% to 89% ± 3%) but significantly decreases the inhibition of β3α6β2α4β2 (*P = 0.0145) and (α4β2)_2_α4 receptor (*P = 0.0482, two-way ANOVA with Tukey's multiple comparison test) (*N* = 3–5, *n* = 3–9). The points are the mean ± S.D.

To begin designing an RVG-derived CPP that is α7 nAChR target-selective, we explored combining regions of *α*-btx lp2 with RVG. We first separated the RVG peptide and *α*-btx lp2 peptide into distinct regions based on protein sequences (Table 1). The RVG peptide contains a sequence in the middle with no homology to the *α*-btx lp2 peptide, so the RVG peptide was broken into three regions (R1, R2, R3) while the *α*-btx lp2 peptide was broken into only two regions (R1, R3). Using TEVC electrophysiology, we determined the apparent potency of each set of synthetic *α*-btx lp2 and RVG fragments by preapplying increasing concentrations of each peptide (0.01–300 µM) for 30s, followed by stimulation with 1s ACh EC_90_ (1300 µM). The full-length *α*-btx lp2 peptide was the most potent (2.5 µM (confidence interval (CI) 2.0, 3.1 µM)), followed by *α*-btx R1 (59 µM (CI 50, 69 µM)) and *α*-btx R3 (462 µM (CI 333, 981 µM)) Figure 1A. The RVG peptide had an enhanced apparent potency (37 µM (CI 28, 42 µM)) when compared to RVG R1, RVG R2, or RVG R3 peptides. RVG R3 was the most potent (176 µM (CI 147, 213 µM)) of the three fragments, with RVG R1 and RVG R2 having minimal or no actions on α7 nAChR function (Figure 1B).

We then combined the regions of RVG and *α*-btx lp2 to construct an α7 nAChR-selective peptide with the goal of maintaining the CPP properties of RVG while enhancing target selectivity (Figure 1C). We hypothesized that the peptide, which contained *α*-btx R1, RVG R2, and RVG R3 (ARR), would likely be the most effective at antagonizing α7 nAChRs, as the *α*-btx R1 and RVG R3 had the most independent inhibitory actions.

The first step in evaluating whether we could generate a potent α7 nAChR subtype selective chimeric peptide was to understand the selectivity of the RVG parent peptide. To this end, the peptide was screened for nAChR subtypes present in the CNS, including α7, α4β2, α3β2, α3β4, concatenated β3α6β2α4β2, α6/α3β2β3, and α4α5β2 (Zoli et al. 2015; Abbondanza et al. 2024). Each isoform of the α4β2, α3β2, and α3β4 subtypes was tested separately for a total of 10 neuronal nAChRs. The RVG peptide is known to antagonize the nAChR response to ACh by competing for the orthosteric binding pocket (O’Brien 2024). We performed a subtype selectivity screen, which allowed us to quickly determine whether the peptides were efficacious on the evaluated nAChRs (Figure 1D). Using TEVC, clamped oocytes expressing the desired nAChR were first exposed to their specific ACh EC_90_ (α4β2α5 12 µM; (α4β2)_2_β2 18 µM; β3α6β2α4β2 26 µM; α6/α3β2β3 40 µM; (α3β2)_2_β2 79 µM; (α3β4)_2_β4 220 µM; (α3β4)_2_α3 410 µM; (α4β2)_2_α4 1.16 mM; α7 1.29 mM; (α3β2)_2_α3 3.05 mM) to provide a baseline response. Following washout of ACh, the oocytes were pre-exposed to 30 s of 100 µM peptide followed by ACh EC_90_ stimulation. This protocol allowed us to quickly determine how much the RVG peptide inhibited each nAChR subtype.

The RVG parent peptide inhibits the ACh response of α7 nAChR the most (83% ± 15%) of any of the tested nAChRs (Figure 1D). The α4β2 subtype is the most abundant nAChR expressed in the CNS (Zoli et al. 1998; Ramachandran Nair and Liu 2019), and both isoforms of α4β2 (HS 27% ± 10%, LS 22% ± 7%), as well as other α4β2-containing subtypes (α4α5β2 30% ± 7% and β3α6β2α4β2 39 ± 12%) ACh-evoked responses were also inhibited by RVG. The remaining subtype ACh-mediated currents were inhibited <26% (α6/α3β2β3 25% ± 6%; (α3β2)_2_β2 15% ± 9%; (α3β2)_2_α3 15% ± 4%; (α3β4)_2_β4 7% ± 3%; (α3β4)_2_α3 12% ± 10%). These results indicate that the RVG peptide inhibits the α7 subtype the most, a key feature we attempted to enhance with our designed chimeric peptides, but also antagonizes the function of other abundantly CNS-expressed nAChRs.

To reduce the probability of off-target effects, we aimed to generate a CPP with high α7 nAChR selectivity by incorporating components of *α*-btx. To screen the three RVG/α-btx chimeric peptides, we used the same protocol as described above for the RVG peptide. The peptide ARR, which is composed of *α*-btx R1, RVG R2, and RVG R3, maximally inhibited α7 nAChR ACh-mediated currents (94% ± 1%) (Figure 1D). However, it also displayed the least selectivity, as five of the 10 nAChRs tested had >50% inhibition (Figure 1D). The RRA (RVG R1, RVG R2, and *α*-btx R3) peptide inhibited heteromeric nAChR ACh-evoked currents <20% (Figure 1D), but only inhibited the α7 nAChR ACh currents by 62% ± 9% (Figure 1D). The remaining ARA (*α*-btx R1, RVG R2, and *α*-btx R3) peptide robustly inhibited α7 nAChR ACh currents (89% ± 3%), while minimally inhibiting the heteromeric subtypes ≤18% (α6/α3β2β3 18% ± 5%; (α3β2)_2_β2 17% ± 4%; (α3β2)_2_α3 18% ± 4%; (α3β4)_2_β4 4.9% ± 0.6%; (α3β4)_2_α3 15% ± 10%). Importantly, the ARA peptide reduced antagonization of the α4β2-containing subtypes (HS 18% ± 2%, LS 10% ± 7%, α4α5β2 20% ± 5%, β3α6β2α4β2 26% ± 6%), demonstrating a significant improvement in α7 nAChR selectivity compared to the RVG peptide (β3α6β2α4β2 *P = 0.0145 and (α4β2)_2_α4*P = 0.0482, two-way ANOVA with Tukey's multiple comparison test).

### The ARA peptide has an improved apparent potency and efficacy

**Figure 2.**
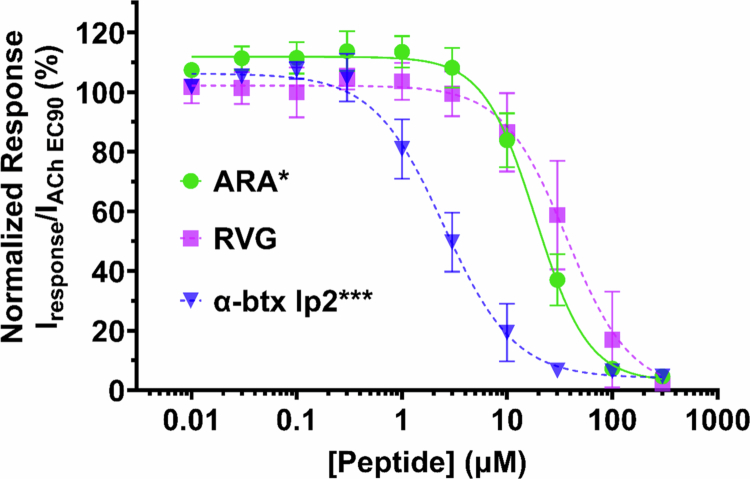
The concentration-response profile for ARA displays an increase in the apparent potency for the α7 nAChR compared to the RVG peptide. Concentration-response profiles of ARA's antagonistic effects on α7 nAChR ACh-evoked responses were generated using TEVC electrophysiology. At each concentration, ARA was pre-applied for 30s, and the α7 nAChRs were activated by a 1s ACh (EC_90_) stimulation. The ARA peptide had a significantly enhanced apparent potency (19 µM (CI 16, 22 µM)) compared to the RVG peptide (37 µM (CI 28, 42 µM)) (*P = 0.0102), but was not as potent as the *α*-btx lp2 peptide (2.5 µM (CI 2.0, 3.1 µM)) (***P = 0.0004, one-way ANOVA with Dunnett's multiple comparison test). Points are the mean ± S.D. (*N* = 3–4, *n* = 5–8).

To determine if the apparent potency of the ARA peptide was enhanced compared to the RVG peptide, as suggested by the selectivity screen, we generated concentration-response profiles using TEVC electrophysiology (Figure 2). As described for RVG and *α*-btx lp2 (Figure 1A,B), concentration-response profiles were generated using α7 nAChR expressing oocytes and 30 s of pre-applied increasing concentrations of the ARA peptide (0.01–300 µM) before ACh EC_90_ (1300 µM) stimulation. The responses were compared to the peptide-naïve ACh-evoked response to normalize the data. The ARA peptide had a significantly enhanced apparent potency (19 µM (CI 16, 22 µM)) compared to the RVG peptide (37 µM (CI 28, 42 µM)) (*P = 0.0102), but was not as potent as the *α*-btx lp2 peptide (2.5 µM (CI 2.0, 3.1 µM)) (***P = 0.0004, one-way ANOVA with Dunnett's multiple comparison test) (Figure 2, Supplemental Table 1).

**Figure 3.**
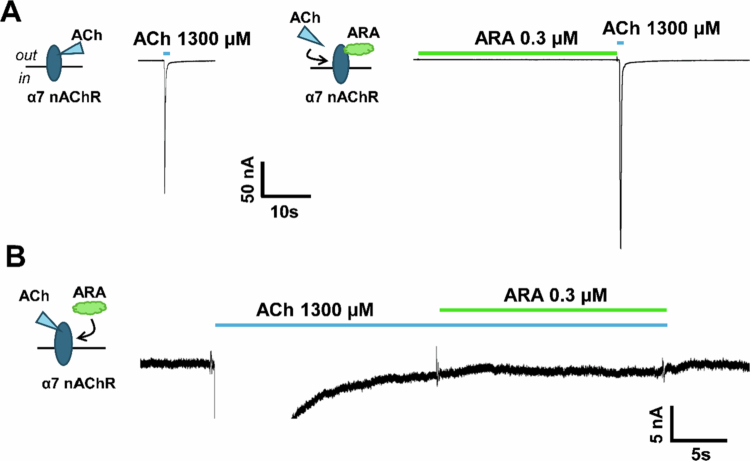
Low concentrations of ARA potentiate ACh responses, but ARA cannot reactivate desensitized α7 nAChRs. α7 nAChR-expressing oocytes were either exposed to 0.3 µM ARA prior to or after ACh application, and responses were recorded using TEVC electrophysiology. Schematics of ligand applications, with the arrow indicating a second ligand applied after the first ligand has bound to α7 nAChR, are shown to the left of each trace. **(A)** Representative trace of the α7 subtype illustrating a 1 s (blue drug bar) ACh-induced baseline response (*left)*. Following a 225 s wash (not shown), 0.3 µM ARA was preapplied for 30 s (green drug bar above trace), and α7 nAChRs were activated by stimulation with 1 s of ACh (*right*). The experiments were repeated *N* = 4, *n* = 5. **(B)** 1300 µM ACh was applied until the α7 nAChR-mediated current was close to baseline and unchanging (~20s). A potentiating concentration of ARA (0.3 µM) was coapplied with ACh to determine whether desensitized α7 nAChRs would be reactivated. No change was observed, demonstrating that ARA is not capable of reactivating desensitized α7 receptors (magnified example trace shown, repeated *N* = 3, *n* = 6).

In Figure 2, the low concentrations of ARA slightly enhanced (114% ± 7%) the ACh EC_90_ response, suggesting that ARA may function as a PAM. Type I PAMs increase the agonist-induced peak current, and type II PAMs, in addition to enhancing the peak response, also slow the rate of receptor desensitization (Sanders and Millar 2023). To investigate whether ARA is a type I or type II PAM, we compared the peptide naïve ACh response to that in which 0.3 µM was preapplied for 30 s, which resulted in an increased peak response (130% ± 10%, Figure 3A). In 2011, Collins et al. conducted a series of experiments demonstrating that PNU−120596 is an α7 nAChR type II PAM that prolongs the receptors' return to baseline and can reactivate desensitized receptors (Collins et al. 2011). Unlike PNU−120596, with application of ARA, there was no significant change in response shape or time to return to baseline. When 0.3 µM ARA was applied to α7 nAChRs during ACh (1300 µM) induced steady-state, no recovery of desensitized receptors was observed (Figure 3B). These data indicate that there may be some slight type I PAM activity of ARA at low concentrations.

**Figure 4.**
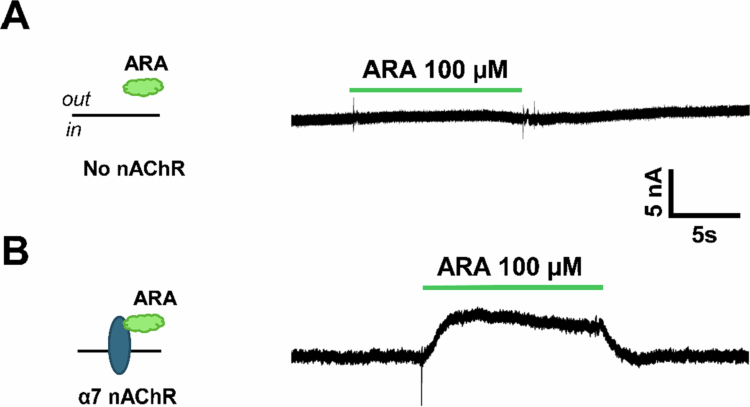
High concentrations of ARA evoke an outward current in the absence of an agonist that is dependent on the α7 nAChR. Uninjected oocyte or α7 nAChR representative responses to ARA captured using TEVC electrophysiology. Schematics of the assay are shown to the left of each trace. (A) Application of 100 µM ARA to an uninjected oocyte caused no response (example trace shown, repeated *N* = 3, *n* = 3). (B) 100 µM ARA produced an outward current in α7 nAChR-expressing oocytes (example trace, *N* = 3, *n* = 3).

At high ARA concentrations (10–300 µM), we observed no effect of ARA application on oocytes not expressing α7 nAChRs (Figure 4A). Interestingly, a small outward current (3–15 nA) was generated when the peptide (10–300 µM) was applied to α7 nAChR-expressing oocytes (Figure 4B). This outward current, coupled with the slight PAM activity (Figure 3A), suggested that the ARA peptide may have silent agonist properties.

**Figure 5.**
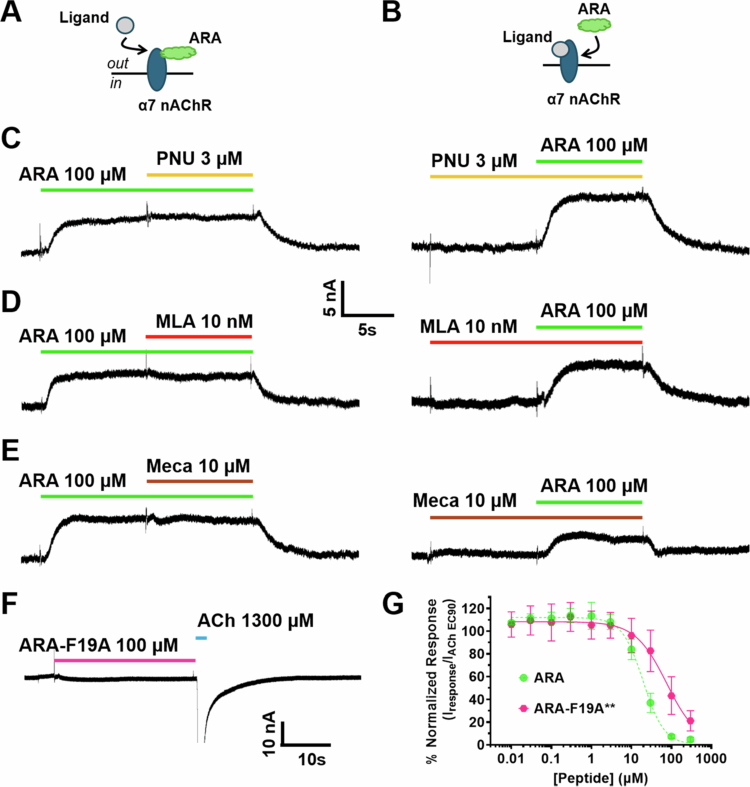
The α7 nAChR ARA-evoked outward current could not be modified by a PAM or antagonists, but could be eliminated with an alanine ARA peptide substitution. α7 nAChR representative responses to PNU−120596, MLA, Meca, and ARA-F19A were captured using TEVC electrophysiology. **(A)** A schematic for traces shown on the left in D–E illustrates that ARA was applied prior to the application of an additional ligand (PNU−120596, MLA, or Meca). **(B)** The experimental outline for the assays shown on the right in C–E shows that PNU−120596, MLA, or Meca were preapplied before the application of ARA. **(C)** The α7 nAChR outward current generated by 100 µM ARA could not be modified by the PAM PNU−120596 after ARA was applied (left) or if PNU−120596 was preapplied (right) (example traces shown, repeated *N* = 3, *n* = 7). **(D)** Postapplication (left) or preapplication (right) of the α7 nAChR competitive antagonist MLA did not prevent or alter the ARA caused outward current (example traces shown, repeated *N* = 3, *n* = 5). **(E)** The noncompetitive channel blocker Meca applied either after (left) or before ARA (right) could also not modify the outward ARA-generated current (example traces shown, repeated *N* = 3, *n* = 6). **(F)** Substituting ARA residue F19 for an alanine eliminated the outward current (magnified example trace shown, repeated *N* = 4, *n* = 4). **(G)** Concentration-response profiles for ARA-F19A demonstrated that it is less potent on α7 nAChRs (76 µM (CI 60, 97 µM)) than ARA does (**P = 0.0084, Welch's unpaired t test). The ARA data are reshown from Figure 2 to facilitate comparison. Points are the mean ± S.D. (ARA-F19A *N* = 4, *n* = 8).

Silent agonists are a class of compounds that, upon binding, induce a conformational change that stabilizes the desensitized state rather than an open state. Previous work with the silent agonist NS6740 has shown that using a type II PAM, such as PNU−120596, can shift the α7 nAChR towards a conducting state without activation (Blunt and Dougherty 2019; Horenstein and Papke 2017). To test whether ARA has silent agonist activity at higher concentrations, we preapplied either ARA (100 µM) or PNU−120596 (3 µM) for 10 s, followed by the coapplication of ARA with PNU−120596 for another 10 s (Figure 5A–C). No receptor activation was observed in either instance, and the outward current created by ARA was unaffected. We next tried to block the outward current with the α7 nAChR competitive antagonist MLA or the noncompetitive antagonist Meca. Neither pre- nor coapplication with either antagonist could mitigate the small outward current created by the application of 100 µM ARA (Figure 5D,E).

In addition to ionotropic activity, the α7 subtype has been shown to activate metabotropic responses by directly interacting with intracellular G-proteins (Nordman et al. 2014; Nordman and Kabbani 2012). Given that the ARA-evoked current is α7-mediated (Figure 4A,B) and that the current generated is slow relative to an ACh response, we reasoned that ARA may trigger activation of the G-protein to activate a distinct effluxing ion channel. We attempted to express the α7(345–348A) mutant, which lacks the G-protein binding capacity (King et al. 2015), in oocytes. Unfortunately, with injecting 20–120 ng of cRNA was injected and waiting 3–14 days post-injection, we were unable to observe the response to ACh (Supplemental Figure 1 A). Next, we attempted to inhibit the endogenous G-protein in oocytes using YM−254890, a known plasma membrane-permeable inhibitor of the Gα protein in the G_q/11_-protein family (Xiong et al. 2019; Xiong 2016). We bath-applied 2–20 µM YM−254890 for 1–24 hour to allow for cell penetration, and regardless of the concentration or incubation time, we were unable to prevent or reduce the outward current generated by ARA (Supplemental Figure 1B). To determine if we could remove the outward current, we substituted the phenylalanine at position 19 for an alanine (ARA-F19A). With the application of 100 µM ARA-F19A, we were able to eliminate the outward current (Figure 5F). Concentration-response profiles demonstrated that while we were able to remove the outward current, this substitution resulted in a fourfold less potent peptide and was thus not used in follow-up experiments (Figure 5G). Future work will include structural ARA modification to eliminate the outward current while maintaining or improving peptide apparent potency.

### ARA is a competitive antagonist

**Figure 6.**
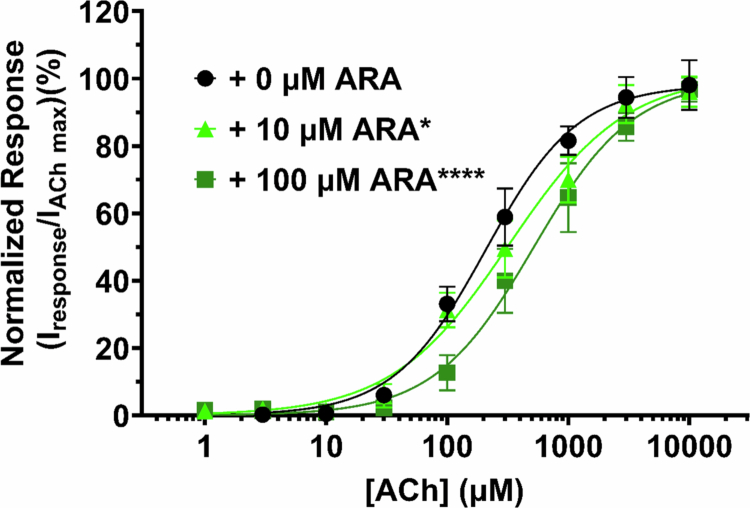
ARA competes with ACh for binding to the α7 nAChR. α7 nAChR-expressing oocytes were coexposed to increasing concentrations of ACh without or with 10 and 100 µM ARA, and responses were observed using TEVC electrophysiology. Coapplication of ARA with increasing concentrations of ACh shifted the response curve to the right, resulting in significant decreases in the apparent potency (10 µM ARA *P = 0.0246 and 100 µM ARA ****P < 0.0001, one-way ANOVA with Dunnett's multiple comparison test). There was no effect on efficacy demonstrating that at inhibiting concentrations, ARA is a competitive antagonist. Points are the mean ± S.D. (*N* = 3–4, *n* = 8–13).

An nAChR competitive antagonist competes with ACh for the orthosteric binding pocket and functionally reduces the ACh apparent potency without changing efficacy (Lentz et al. 1987; Ho et al. 2020; Harvey and Luetje 1996). As we have previously determined that RVG is a competitive antagonist, we hypothesized that ARA antagonizes α7 nAChRs by the same mechanism (O’Brien 2024). To this effort, we coapplied increasing concentrations of ACh (1 µM–10 mM) with 0 µM, 10 µM, or 100 µM ARA and normalized each response to a 10 mM ACh response without ARA present (Figure 6). Applying either 10 or 100 µM ARA resulted in a rightward shift in the concentration response curve compared to ACh without ARA coapplication (+0 µM ARA IC_50_: 208 µM (CI 181, 240 µM), +10 µM ARA: 322 µM (CI 271, 390 µM), and +100 µM ARA: 518 µM (CI 445, 611 µM), Supplemental Table 2). While significant changes in apparent potency were observed (+10 µM ARA *P = 0.0204 and +100 µM ARA ****P < 0.0001, one-way ANOVA with Dunnett's multiple comparison test), there was no change in efficacy with ARA coapplication. These data are consistent with ARA inhibiting α7 nAChRs via competitive antagonism.

### ARA is not cytotoxic

**Figure 7.**
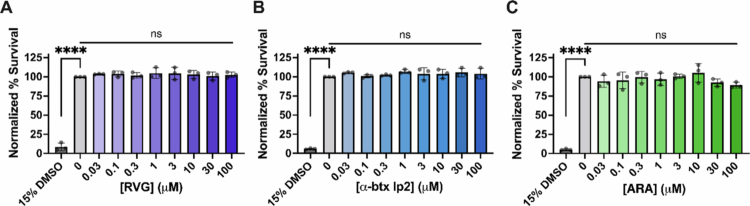
RVG, *α*-btx lp2, and ARA peptides are not cytotoxic at functional concentrations. Cytotoxicity profiles for (A) the RVG parent peptide, (B) the *α*-btx lp2 peptide, and (C) ARA using α7 nAChR-transfected N2a cells. Peptides (0.03–100 μM) that were preapplied for 24 hour and assessed by the alamarBlue Cell Viability Assay show no significant cytotoxic effects (one-way ANOVA with Tukey's multiple comparison test, ****P < 0.0001). 15% DMSO was the positive control. Points are the mean ± S.D. (*N* = 3, *n* = 6–9).

Peptide therapies are attractive, partially due to their low cytotoxicity profile (Wang 2022). However, a subset of CPPs, specifically those designed to selectively target and eliminate cancerous cells, are cytotoxic (Maraming 2018; Wang et al. 2025). In efforts to develop ARA as a potential drug-delivery CPP, we performed a comprehensive cytotoxicity screening using N2a cells transfected with the α7 subunit DNA as a preliminary look at the potential safety profile. We used alamarBlue cell viability reagent and found that after 24 hour of incubation with 0.03–100 µM of RVG, *α*-btx lp2, or ARA peptides, no cytotoxic effects were observed (^ns^*P* > 0.05, one-way ANOVA with Tukey's multiple comparison test) (Figure 7 and Supplemental Figure 2). A 15% DMSO control was used to demonstrate positive detection of dead cells (****P < 0.0001, one-way ANOVA with Dunnett's multiple comparison test, with the untreated group as a control).

### ARA preferentially labels N2a cells expressing α7 nAChRs

Some drug delivery studies have shown that RVG derivatives, such as RVG_29_, are capable of delivering cargo, including molecules and siRNAs, into the CNS and only require concentrations in the high nanomolar to low micromolar range (Jia 2021; Kumar 2007). We next aimed to determine whether ARA selectively targets only α7 nAChRs by using the mammalian N2a cell line with a neuronal phenotype. Each peptide was tagged with a fluorophore: ARA was labeled with FITC (ARA-FITC), full-length *α*-btx was conjugated with AF648 (*α*-btx-AF648), and RVG was bound to FITC (RVG-FITC). α7 nAChR-overexpressing N2a cells were incubated for 24 hour with either *α*-btx-AF647, RVG-FITC, or ARA-FITC peptides.

**Figure 8.**
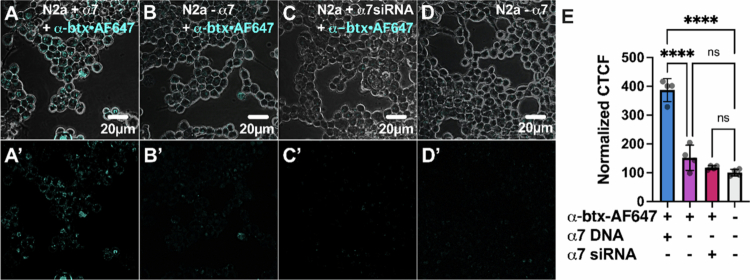
Demonstration of α7 nAChR-specific labeling with *α*-btx-AF647. Confocal images of (A) α7 nAChR-transfected, (B) nontransfected, and (C) α7 nAChR siRNA-transfected N2a cells treated with 80 nM *α*-btx-AF746 for 24  hour show the selective binding of *α*-btx-AF647 to cells overexpressing α7 nAChRs and little binding to cells with residual endogenous expression. (D) Untreated N2a cells were imaged in the AF647 fluorescence channel. Α-btx-AF647 selectively interacts with cells expressing α7 nAChRs and interacts little with cells not transfected with α7 nAChRs. (A’–D’) Same as (A-D) but without the phase channel. Quantification of CTCF (E) demonstrates high fluorescence in cells expressing α7 nAChRs, with no significant fluorescence in non-transfected and α7 nAChR siRNA-transfected cells (one-way ANOVA with Tukey's multiple comparison test, *N* = 4, *n* = 120–174). The values are the means ± S.D. and were normalized to those of untreated controls (D).

Live-cell confocal microscopy showed robust *α*-btx-AF647 (80 nM) binding to α7 nAChR DNA-transfected cells (Figure 8A,D). Conversely, non-transfected cells displayed minimal labeling (Figure 8B) relative to non-labeled cells (Figure 8D). To verify that the slight increase in labeling using non-transfected cells could be attributed to endogenous α7 nAChR expression, cells were transfected with α7 siRNA and treated with *α*-btx-AF647 24 hour later. α7 siRNA-treated cells had less *α*-btx-AF647 labeling (Figure 8C) than non-transfected treated cells, and were not different from untreated cells (Figure 8D). Quantification of fluorescence as CTCF (Figure 8E) verifies the highest *α*-btx-AF647 binding in α7 nAChR DNA-transfected cells (386.7% ± 39.8%, ****P < 0.0001, one-way ANOVA with Tukey's multiple comparison test), while non-transfected and siRNA-transfected cells show only low levels of CTCF (151.6% ± 43.9%, ^ns^*P* = 0.1298, and 117.6% ± 7.2%, ^ns^*P* = 0.8, respectively), comparable to background fluorescence levels in untreated cells (100% ± 11.7%). This emphasizes that the binding of *α*-btx-AF647 was due to the presence of α7 nAChRs.

**Figure 9.**
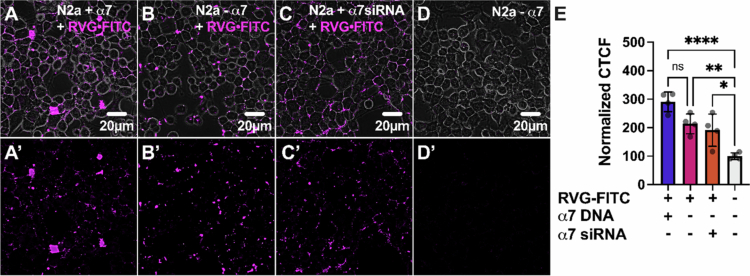
RVG CPP nonselectively interacts with N2a cells. Confocal images of **(**A) α7 nAChR-transfected, (B) non-transfected, and (C) α7 nAChR siRNA-transfected N2a cells treated with 30 μM RVG-FITC for 24 hour showing RVG binding to N2a cells regardless of α7 nAChR presence. (D) Untreated N2a cells imaged in the FITC fluorescence channel. RVG interaction with N2a cells is not dependent on the expression of the α7 nAChR. (A’–D’) Same as (A-D) but without the phase channel. Quantification of CTCF (E) confirmed similar fluorescence levels in cells with and without α7 nAChRs (one-way ANOVA with Tukey's multiple comparison test, *N* = 4, *n* = 120–135). The values are the means ± S.D. and were normalized to those of untreated controls **(D)**.

As shown in Figure 1D, RVG appears to be mildly selective for the α7 nAChR. However, the rabies virus glycoprotein targets other host proteins, including neural cell adhesion molecule (Kadmon et al. 1990; Thoulouze et al. 1998), p75 neurotrophin receptors (Bai et al. 2008), metabotropic glutamate receptor subtype 2 (Wang 2018), and integrin β1 (Shuai 2020), which are all expressed in N2a cells. We thus anticipated that the application of RVG-FITC would result in the labeling of the abovementioned RVG targets, in addition to α7 nAChRs. α7 nAChR over-expressing N2a cells were treated with 30 µM of RVG-FITC, and the resulting fluorescence was visualized using live-cell confocal microscopy. The cells overexpressing α7 nAChRs showed the highest RVG-FITC-associated fluorescence (Figure 9A). However, cells that were not α7 nAChR transfected (Figure 9B) or had been treated with α7 siRNA (Figure 9C) showed elevated levels of fluorescence as well, compared to non-treated cells, demonstrating substantial non-α7 nAChR binding of the RVG-FITC peptide (Figure 9D). Quantification of RVG-FITC CTCF (Figure 9E) confirmed a significant increase in fluorescence in α7 nAChR-transfected cells relative to blank cells, nearly tripling the CTCF value (290.7% ± 34.5%, ****P < 0.0001, one-way ANOVA with Tukey's multiple comparison test). In contrast, nontransfected (213.4% ± 34.9%, **P = 0.0056) and α7 siRNA-transfected (191.1% ± 56.7%, *P = 0.0237) cells exhibited less pronounced increases in CTCF, but were still significantly more prevalent than background (100% ± 11.4%). These results align with previous findings, substantiating that RVG utilizes nAChRs for interaction with cells (O’Brien 2024; Lian et al. 2022; Hueffer 2017) but does not rely solely on the α7 nAChR subtype.

**Figure 10.**
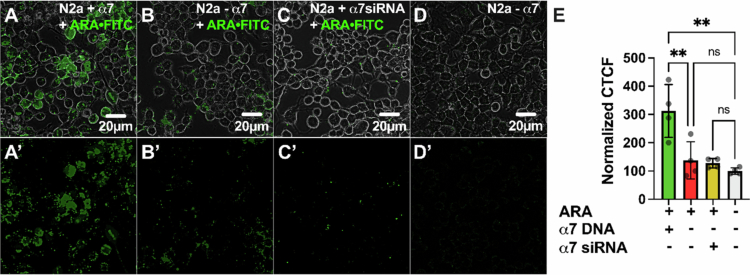
ARA preferentially interacts with cells expressing α7 nAChRs. Confocal images of (A) α7 nAChR-transfected, (B) nontransfected, and (C) α7 nAChR siRNA-transfected N2a cells treated with 30 μM ARA-FITC for 24 hour showing high fluorescence levels associated with ARA-FITC in cells expressing α7 nAChRs. (D) Untreated N2a cells were imaged in the FITC fluorescence channel to account for autofluorescence. Endogenously expressed α7 nAChRs in N2a cells contribute to the staining of nontransfected N2a cells, which is reduced by the transfection of α7 siRNA. (A’- D’) Same as (A-D) but without the phase channel. The quantification of CTCF (E) demonstrates significantly more fluorescence in cells expressing α7 nAChRs (one-way ANOVA with Tukey's multiple comparison test, *N* = 4, *n* = 117–126). The values are the means ± S.D. and were normalized to those of the untreated control (D).

As the ARA peptide is a chimeric peptide with components of the α7 nAChR-selective *α*-btx and the nonselective RVG peptide, we tested ARA selectivity using α7 nAChR over-expressing N2a cells as performed for the two parent peptides. ARA-FITC fluorescence labeling was visualized via live-cell confocal microscopy. Cells expressing the α7 nAChR exhibited pronounced labeling with 30 µM ARA-FITC 24 hour postexposure (Figure 10A). In contrast, nontransfected cells displayed minimal fluorescence (Figure 10B), and siRNA knockdown of endogenous α7 nAChRs diminished the ARA-FITC labeling (Figure 10C) as seen in the nontransfected, nontreated groups (Figure 10D). These data support that α7 nAChR is responsible for robust ARA-FITC labeling. Quantitative analysis of fluorescence intensity (Figure 10E) revealed a substantial increase in ARA-FITC-associated fluorescence in α7 nAChR-transfected cells (312.3% ± 93.2%, **P = 0.0011, one-way ANOVA with Tukey's multiple comparison test) in comparison to blank cells (100% ± 11.4%). ARA-FITC labeling was drastically reduced in non-transfected cells (137.7% ± 66.0%, ^ns^*P* = 0.8) and α7 siRNA-treated cells (127.6% ± 16.8%, ^ns^*P* = 0.9), which were not significantly different from untreated cells.

### The ARA peptide is a CPP

Prior studies have demonstrated the ability of RVG-derived peptides to penetrate cultured cells, neurons, and even the brains of mice and rats by a receptor-mediated mechanism (Jia 2021; Kumar 2007; O’Brien 2024). Our chimeric peptide, ARA, incorporates segments of the neurotoxin *α*-btx, introducing a novel structural aspect that could hinder cellular entry.

**Figure 11.**
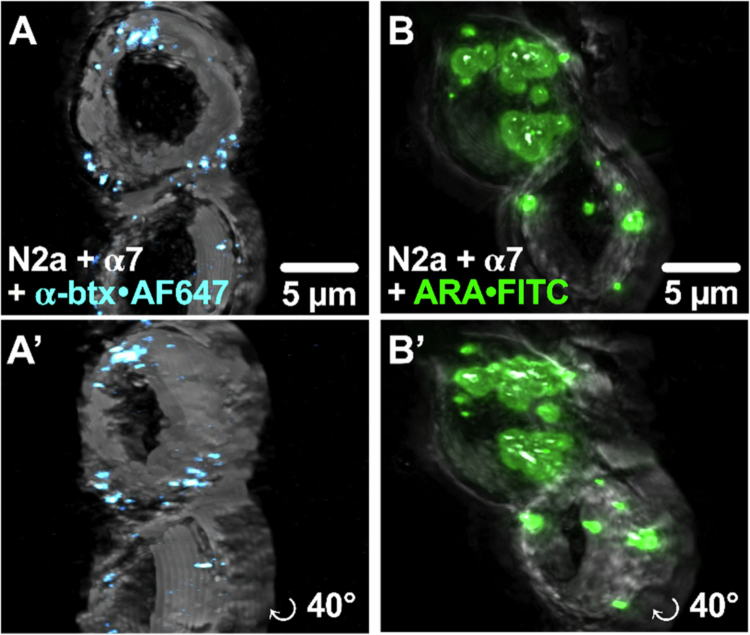
ARA transports FITC cargo into α7 nAChR-expressing cells. Representative 3D projections from z-stack confocal images of α7 nAChR-transfected N2a cells (z-stack slice size: 1 μm) treated with (A) 30 μM FITC-tagged ARA peptide or (B) 40 nM *α*-btx-AF647 for 24 hour prior to imaging. (A’ and B’) Rotated around the y-axis by 40°. ARA appears in the cell interior, while *α*-btx binds to the cell surface without internalization.

To assist with visualization of the location of the fluorescence, N2a cells expressing α7 nAChRs were treated with *α*-btx-AF647 (40 nM) or ARA-FITC (30 µM) for 24  hr prior to imaging. Z-stack images were captured and compiled to create 3D projects (Figure 11 and Supplemental Videos 1 and 2). Upon careful inspection of the live-cell confocal images, we observed very different fluorescence staining patterns of *α*-btx-AF647 and ARA-FITC (Figure 11). *α*-btx-AF647 fluorescence occurred mostly around the perimeter of the cells (Figure 11A). In comparison, ARA-FITC fluorescence was located throughout the interior, demonstrating that our ARA peptide was inside the cells (Figure 11B). It has been shown for multiple other RVG derivatives that nAChRs are crucial interaction scaffolds for these peptides to connect to the cellular plasma membranes and use these receptors as vehicles to internalize, likely through receptor-mediated endocytosis (Gong et al. 2012; You 2018; Liu 2009). Given the retention of some RVG structures within ARA, it appears that the ARA peptide employs α7 nAChRs to enter the cell a receptor-mediated process.

## Discussion

In this study, we developed and characterized a novel chimeric CPP created through combining the regions of *α*-btx, which is responsible for α7 nAChR targeting, and the rabies virus glycoprotein region, which is hypothesized to mediate cell entry. ARA is highly selective for the α7 nAChR subtype, unlike the parent RVG peptide, with good apparent potency at low micromolar concentrations (Figures 1 and 2). ARA has multiple functional modalities for the α7 subtype. At low concentrations (<3 µM), ARA potentiates ACh-induced currents in a modest amount. At higher concentrations (>3 µM), ARA is a competitive antagonist, attenuating the ACh-induced currents and shifting the ACh apparent potency to the right (Figure 6). Curiously, >10 µM of ARA not only reduced the ACh response but also induced a slow outward current that cannot be removed with other antagonists (Figure 5). Attempts to determine if the outward current generated by ARA application was due to activation of a G-protein-mediated signaling cascade were unsuccessful. Through modifying the ARA peptide by an F19A substitution, we were able to eliminate the outward current at the cost of reducing apparent potency (Figure 5F,G). Future work to improve ARA will include structural refinements to eliminate the production of an outward current while maintaining or improving the apparent potency.

Using α7 nAChR-expressing N2a cells, a mouse-derived cell line with a neuronal phenotype, we performed an initial toxicity screen and found that ARA is not cytotoxic up to the highest concentration tested (100 µM) (Figure 7). We demonstrate a clear α7 nAChR dependence for the cellular interaction of ARA in both our electrophysiology (Figure 1) and cell culture assays (Figure 10). Using cultured cells, ARA-FITC substantially labeled cells expressing α7 nAChRs, with nominal labeling observed in cells with endogenous α7 nAChR expression (Figure 10). These findings suggest that the ARA peptide requires the presence of α7 nAChRs on the cell surface to bind to N2a cells. To further accentuate the importance of α7 nAChRs for cellular interaction of ARA, we used α7 nAChR-siRNA to knock down receptor expression. The cells subjected to α7 nAChR knockdown exhibited the least amount of fluorescent labeling in response to peptide treatment (Figure 10E). Comparison with the α7 nAChR-selective antagonist *α*-btx revealed analogous interaction profiles, highlighting the enhanced selectivity of ARA over the less-selective RVG peptide (Figures 8 and 9). These findings further establish that our ARA chimeric peptide exhibits enhanced selectivity for α7 nAChRs as targets on neuronal-like cells, displaying minimal non-selective binding *in vitro* in comparison to the RVG parent peptide. Visual observations were substantiated by quantitative analysis of fluorescence values associated with FITC tags, providing compelling evidence that α7 nAChRs are crucial for the interaction of ARA with neuronal-like cells.

RVG_29_, a CPP that can transport therapeutic cargo into the mouse brain when given peripherally, has proven not to be cytotoxic, and multiple doses given over weeks are well tolerated (Jia 2021; Kumar 2007; Cao 2024). Given that ARA is more target-selective than RVG is (Figure 1D), it is plausible that the concentrations required for *in vivo* drug delivery may be even lower than those currently used with RVG_29_. This is an important and exciting area of research for future efforts. Owing to the lack of cytotoxic effects, ARA is a promising candidate for further investigation in drug delivery, highlighting its potential for safer and more effective therapeutic interventions.

One of our most exciting findings is that ARA is an α7 nAChR target-selective CPP. Visualization through 3D projections revealed fluorescence in the intracellular space, indicating that, like RVG_29_, ARA shares the capability to enter and transport cargo, including FITC, into cells (Figure 11B). Receptor-mediated endocytosis, as proposed for RVG_29_, is a potential mechanism for how ARA gains access to the inside of the cell (Gong et al. 2012; You 2018; Liu 2009). It remains to be determined the cargo size limitation ARA can transport across the cell membrane, and if the cargo can escape the endosome.

Certain receptors, including the α7 subtype, are expressed on intracellular organelles, including the mitochondria (Nakamura et al. 2025; Gergalova 2012; Hayashi and Su 2007; Bénard 2012). In the outer mitochondrial membrane of microglia, α7 nAChRs appear to regulate Ca^2+^ accumulation and cytochrome c release, the initial step of apoptosis induction (Gergalova 2012), and choline-stimulated α7 nAChR promotes ATP production (Nakamura et al. 2025). Therefore, it is possible that ARA could be beneficial not only for targeting plasma membrane-expressed α7 nAChRs, but also those expressed internally. Future development of therapies that are intended to target mitochondria expressing the α7 nAChR could incorporate a macromolecule-selective CPP, like ARA, to deliver a therapeutic payload into the cytosol and subsequently into the mitochondria.

Nonselective CPPs have been used to transport cargo across *in vitro* models of the BBB and into the brain *in vivo* using mice (Song 2005; Ghorai et al. 2023; Chiu et al. 2004). RVG_29_, attached to nine unnatural arginine amino acids (9dR), has been used to transport several types of siRNA across the mouse BBB when injected into the tail vein (Jia 2021; Kumar 2007). However, RVG peptides have been demonstrated not to be target-directed, which limits their therapeutic potential by generating off-target effects (Figures 1 and 9) (O’Brien 2024). A commonly overlooked feature of drug delivery systems is the functional effect of the CPP on the cellular target(s). An additional effort could be to engineer an agonistic α7 nAChR-selective CPP. Future work developing ARA, or derivatives, as an α7 nAChR-selective therapeutic delivery system could have applications for the treatment of neurological conditions involving the α7 nAChR, including Alzheimer's disease and major depressive disorder (Mineur et al. 2018; Fernandes 2018; Ren et al. 2020; Martin et al. 2004).

ARA has multiple modalities for α7 nAChRs, including potentiation and antagonism, depending on the concentration, which could be leveraged for clinical applications. For example, during early stages of Alzheimer's disease, α7 nAChRs are upregulated in response to chronic exposure to amyloid-β_1-42_ (Aβ), which may contribute to hyperexcitability (Liu et al. 2013; Dineley et al. 2002; Rennie 2025). The excessive intracellular Ca^2+^ associated with hyperexcitability stimulates excitotoxic signaling cascades and neuronal loss. In primary hippocampal neurons, pretreatment of MLA prevented this hyperexcitability (Liu et al. 2013). During the early stages of AD, it may be beneficial to treat patients with a high inhibiting dose of ARA complexed with another payload to dampen the α7 nAChR activity and deliver an additional therapeutic. However, in later stages of the disease, the α7 nAChR is downregulated (Oddo 2005). It may be beneficial to deliver a potentiating concentration of ARA to enhance the cholinergic activity and deliver a payload, or to use a different therapeutic during this disease state. The dynamic relationship between α7 nAChR expression and AD stage makes it challenging to effectively target an efficacious therapy to the desired cells at the most beneficial time. It would be advantageous to have a fleet of target-selective α7 nAChR CPPs that have a range of effects on the receptor, including agonism, positive allosteric modulation, and antagonism. These tools could potentially be beneficial in the treatment of neurological conditions involving the α7 nAChR, including neuroinflammation, neuropathic pain, schizophrenia, and AD (Magnussen 2025; Luo and Huang 2024).

## Conclusions

The development of CPPs as drug-delivery tools is a significant advancement in enabling the potential transport of therapeutic cargo into the brain. CPPs offer a promising strategy for overcoming the BBB, which has long been a challenge in treating neurological disorders. Here, we have presented the generation of ARA, a novel, potent, and highly target-selective α7 nAChR CPP. CPPs typically have lower binding affinities than antibodies but less potential for immunogenicity, susceptibility to proteases due to their limited size (<30 amino acid residues), stronger penetration ability, easier synthesis and modification, and lower production costs (Todaro et al. 2023; Liu et al. 2021). These positive attributes of peptides make CPPs active therapeutic agents.

Clinically relevant applications of ARA include attaching siRNA unnatural arginine (9dR) hook, or cargo-loaded nanoparticles or exosomes, as has been accomplished using RVG_29_ (Jia 2021; Kumar 2007; Chen 2024; Han 2025). These modifications allow for the potential improvement in delivering a variety of therapeutics to cells expressing α7 nAChRs. Critically, high target selectivity of ARA ensures that the therapeutic payload is delivered to specific cell types or tissues, enhancing the therapeutic efficacy while minimizing the off-target effects. For drug delivery systems to be effective and safe, they must exhibit low cytotoxicity to avoid damaging healthy cells and tissues, a property that ARA may also possess based on our cell assays. The combination of these factors makes ARA an exciting and promising approach for advancing targeted therapies. The presented data serve as a foundation for future investigations, including *in vivo* testing, to validate and further explore the potential of ARA as an efficient drug delivery agent for intracellular and CNS-targeted applications.

## Supplementary Material

Supplementary material Supplemental material

Supplementary material Supplemental Figures

## Data Availability

The data presented in this study are available upon request from the corresponding author.
