# Supplementary material for: Development of a novel alpha7-nicotinic acetylcholine receptor-selective cell-penetrating peptide for intracellular cargo transport
Source: Drug Deliv. 2025 Nov 30;32(1):2587378. doi: 10.1080/10717544.2025.2587378 (PMC12667308; doi:10.1080/10717544.2025.2587378)
Supplement: Supplementary material — Supplemental material [file IDRD_A_2587378_SM7152.docx]

**Supplemental Table 1. Quantified data for the peptide pre-application α7 nAChR inhibition concentration-response profiles.** Pre-application peptide concentration response data were fit using non-linear curve fitting. RVG R1 and R2 potency and N_H_ values could not be determined (N.D.) as these peptides minimally inhibited the α7 subtype. A potency was determined for α-btx R3, but the value is unreliable due to the minimal antagonistic actions at high concentrations (300 µM), resulting in a poor curve fit of the data (R^2^ = 0.4783). Therefore, we chose not to include α-btx R3 in the statistical analysis. Data are presented in Figures 1 and 2.

| **Peptide Treatment** | **N (n)** | **IC_50_ (μM)**  **(95% CI)** | **N_H_**  **(95% CI)** |
| --- | --- | --- | --- |
| α-btx R1 | 4 (6) | 59  (50 – 69) | -1.6  (-1.3 to -2.1) |
| α-btx R3 | 4 (7) | 462  (333 – 981) | -1.3  (-0.7 to -2.6) |
| α-btx lp 2 | 3 (5) | 2.5  (2.0 – 3.1) | -1.2  (-1.0 to -1.4) |
| RVG R1 | 4 (7) | N.D. | N.D. |
| RVG R2 | 4 (6) | N.D. | N.D. |
| RVG R3 | 3 (5) | 176  (147 – 213) | -1.2  (-0.9 to -1.4) |
| RVG | 4 (8) | 37  (28 – 42) | -1.5  (-1.2 to -1.9) |
| ARA | 4 (5) | 19  (16 – 22) | -1.7  (-1.4 to -2.0) |

**Supplemental Table 2. Calculated parameters for ARA competitive antagonist Ach concentration-response profiles.** Non-linear curve fitting was used to analyze the data, and are shown in Figure 5.

| **Co-application ACh concentration response profiles** | | | |
| --- | --- | --- | --- |
| **Peptide Treatment** | **N (n)** | **EC_50_ (μM)**  **(95% CI)** | **N_H_**  **(95% CI)** |
| none | 3 (8) | 208  (181 – 240) | 1.14  (1.01 – 1.29) |
| 10 µM ARA | 4 (12) | 322  (271 – 390) | 0.90  (0.81 – 1.00) |
| 100 µM ARA | 4 (13) | 518  (445 – 611) | 1.06  (0.95 – 1.18) |

**Supplement Figure 1: The ARA outward current could not be prevented by blocking metabotropic G-protein signaling.** Schematics of the assays are shown above each representative trace. **(A)** Example trace of an oocyte injected with 40 ng of α7(345 - 348A) nAChR cRNA and tested five days post-injection exhibited no response to 1300 μM ACh application. Similar experiments testing a range of cRNA concentrations and recording from 3 - 14 days post-cRNA injection all resulted in no ACh-evoked responses (N = 4, n = 12). **(B)** Example trace of an α7 nAChR expressing oocyte exposed to a 20 μM YM-254890 solution for 2hr. Following 100 μM ARA was applied, and the resulting outward current was unaffected by YM-254890 exposure (N = 2, n = 5).

**Supplement Figure 2: RVG, α-btx lp2, and ARA show no cytotoxicity at functional concentrations in non-transfected cells.** Cytotoxicity profiles for **(A)** the RVG parent peptide, **(B)** the α-btx lp2 peptide, and **(C)** ARA on non-transfected N2a cells assessed by the alamarBlue Cell Viability Assay (One-way ANOVA with Tukey’s multiple comparison test, ****P < 0.0001). Points are the mean ± S.D. (N = 3, n = 6 - 9).

**Supplement Video 1:** 3D movie animation of α-btx-AF647-labeled N2a cells transfected with α7 nAChR and NACHO DNA, showing binding of α-btx-AF647 to the plasma membrane of cells. Note that α-btx-AF647 fluorescence is not found in the interior of the cells. The video can be found at dx.doi.org/10.6084/m9.figshare.29922584.

**Supplement Video 2:** 3D movie animation of ARA-FITC-labeled α7 nAChR expressing N2a cells. Strong ARA-FITC fluorescence can be inside the cells. The video can be found at dx.doi.org/10.6084/m9.figshare.29922584.
