# Supplementary figures and images for: Development of a novel alpha7-nicotinic acetylcholine receptor-selective cell-penetrating peptide for intracellular cargo transport
Source: Drug Deliv. 2025 Nov 30;32(1):2587378. doi: 10.1080/10717544.2025.2587378 (PMC12667308; doi:10.1080/10717544.2025.2587378)

**Supplemental Figure 1**


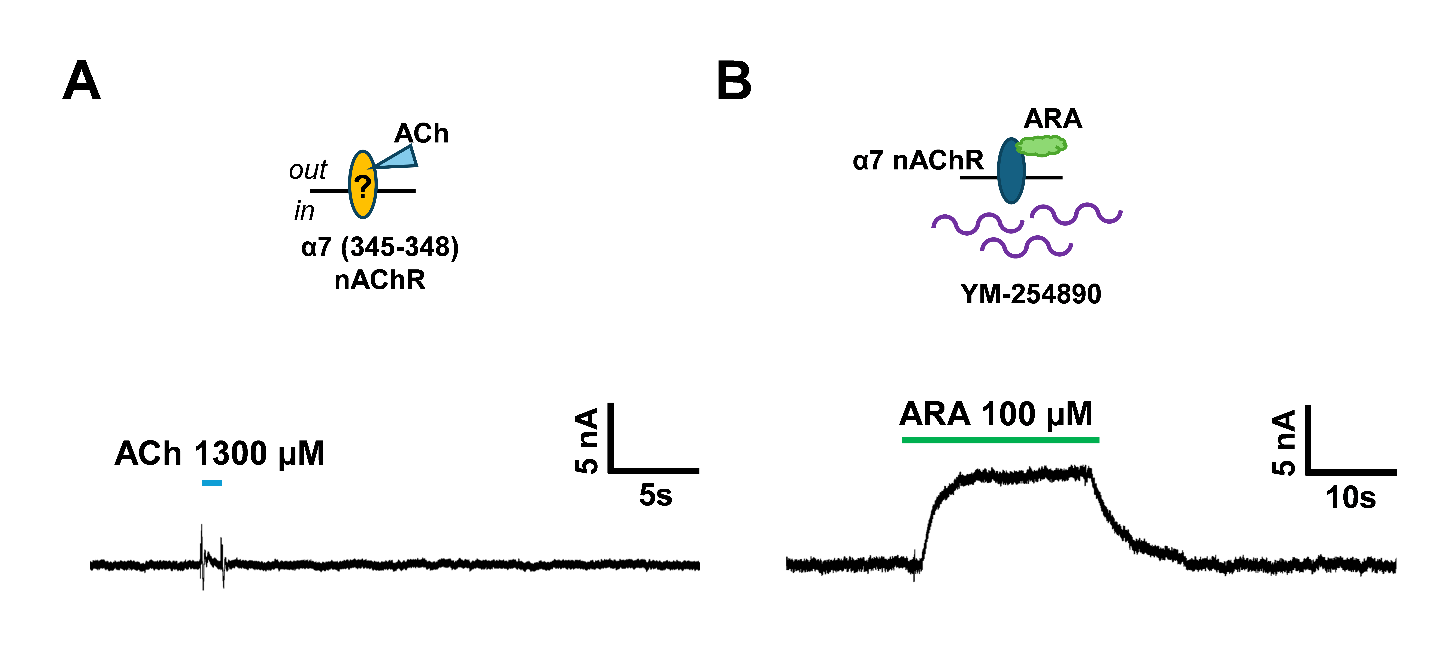


**Supplemental Figure 2**


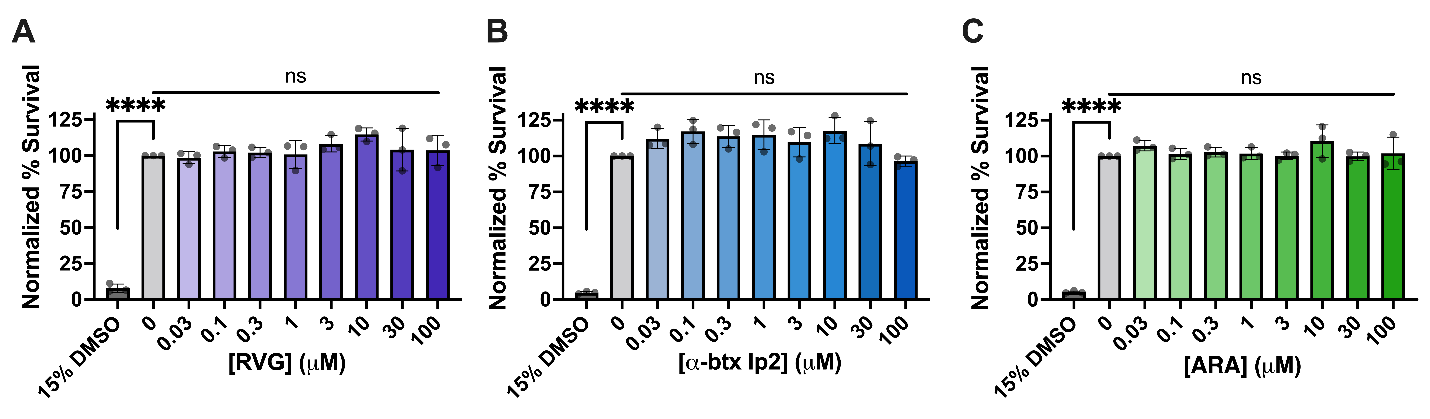

Supplement: Supplementary material — Supplemental Figures [file IDRD_A_2587378_SM7151.docx]
